# Surgical Treatment of Pancreatic Ductal Adenocarcinoma

**DOI:** 10.3390/cancers13081971

**Published:** 2021-04-20

**Authors:** Kongyuan Wei, Thilo Hackert

**Affiliations:** Department of General, Visceral and Transplantation Surgery, University of Heidelberg, Im Neuenheimer Feld 420, 69120 Heidelberg, Germany; jasonwky@163.com

**Keywords:** pancreatic ductal adenocarcinoma, surgical treatment, technical advances

## Abstract

**Simple Summary:**

Surgery is the only potential cure for pancreatic ductal adenocarcinoma and should always be combined with adjuvant chemotherapy or other multimodal treatment. Besides the advances in such multimodal approaches, there has been substantial progress in surgical techniques to especially address advanced resections. These techniques include specific operative steps, such as ‘artery first’ or ‘uncinate first’ approaches as well as techniques that allow safe vascular resection and reconstruction to achieve radical tumor removal. Most recently, also minimally-invasive and robotic approaches have been adopted for pancreatic cancer surgery; however, there is no high-level evidence on these evolving techniques especially with regards to long-term results compared to conventional surgical techniques.

**Abstract:**

Pancreatic ductal adenocarcinoma (PDAC) represents an aggressive tumor of the digestive system with still low five-year survival of less than 10%. Although there are improvements for multimodal therapy of PDAC, surgery still remains the effective way to treat the disease. Combined with adjuvant and/or neoadjuvant treatment, pancreatic surgery is able to enhance the five-year survival up to around 20%. However, pancreatic resection is always associated with a high risk of complications and regarded as one of the most complex fields in abdominal surgery. This review gives a summary on the surgical treatment for PDAC based on the current literature with a special focus on resection techniques.

## 1. Introduction

Pancreatic ductal adenocarcinoma (PDAC) still remains a big therapeutic challenge for its poor prognosis and will likely becomes the second cause of cancer death within the next decade [1,2]. Although there are mounts of advanced treatments including adjuvant chemotherapy, surgical therapy is always regarded as the most effective one to attain the long-term survival for the patients with PDAC [3]. Unfortunately, less than 20% of patients with pancreatic cancer are considered as the surgically resectable cases until now [2]. Additionally, most of the patients with metastatic disease are not suitable for resection according to the safety and efficacy affected by the historical concerns [4]. However, owing to the development of systematic chemotherapy and improvement of surgery, extended indications of the pancreatic resection are applied in clinical practice, including technical advancements as well as patient criteria such as advanced age [5].

### 1.1. Definition of Resectability

**Figure 1:**
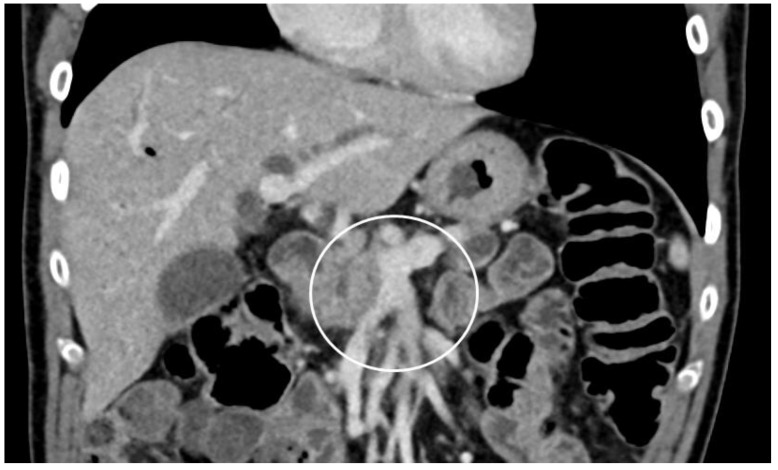
Anatomical borderline resectability, contrast enhanced CT scan, and coronary reformatting. Pancreatic head cancer with contact to superior mesenteric vein/portal vein confluence (white circle), vascular reconstruction technically possible.

There are various classifications reported for the differentiation of resectable, borderline-resectable, and unresectable pancreatic cancers [6,7,8,9,10,11]. The definition of resectability is made mainly based on scientific associations as well as the MD Anderson Classification [8,10]. The AHPBA/SSO/SSAT Classification was modified by the National Comprehensive Cancer Network (NCCN) further as well as the International Study Group of Pancreatic Surgery (ISGPS) [11,12]. Therefore, resectability now is classified by the invasion of important adjacent vessels, especially referring to the celiac trunk, superior mesenteric artery (SMA), and the portal (PV) or superior mesenteric vein (SMV). Pancreatic cancer is regarded as resectable if there are no major vessels involved. Borderline resectable pancreatic cancer is defined as a pancreatic cancer with involvement of the portal vein and/or superior mesenteric vein and the involved segments of vessels allow resection and reconstruction (Figure 1).

Furthermore, if the superior mesenteric artery or the celiac trunk are invaded, pancreatic tumors are considered as locally advanced and unresectable, however arterial resections and reconstructions can be performed by experienced surgeons. Actually, for determination of resectability, the relationship between the tumor and mesenteric/hepatic vessels is the critical topic to obtain R0 resection [13,14,15]. Hence, through preoperative staging and imaging, pancreatic tumors are divided into four types: resectable, borderline resectable, locally advanced and metastatic. Currently, upfront surgery is recommended in resectable pancreatic cancer [7,11,12]. In contrary to the surgical and anatomical considerations to evaluate resectability, the International Association of Pancreatology developed a more comprehensive definition of resectability using three different factors: 1. anatomical; 2. biological; 3. conditional [7]. For anatomical criteria it basically includes the above mentioned factors and basically a serum carbohydrate antigen (CA) 19-9 level more than 500 U/mL or regional lymph node metastases diagnosed by biopsy or positron emission tomography-computed tomography. Potentially resectable disease based on anatomic criteria is transferred to borderline resectability if these factors are present. Conditional factors include the ECOG classification of patients and may also shift potentially resectable disease based on anatomic and biologic criteria towards a borderline resectable status if classification equals or exceeds ECOG 2 [7]. The detailed definition of anatomical resectability is displayed in Table 1. Recently, the BACAP Consortium published a BACAP Score to predict the resectability of pancreatic adenocarcinoma based on anatomical considerations (vascular thrombosis, tumor localization, tumor size) as well as conditional evaluation (WHO performance status) and symptoms (pain, weight loss) [16]. Based on the analysis of a prospectively collected 814-patient cohort, this score will be evaluated in further clinical trials.

Nowadays, CT, MRI, and PET are applied in the imaging detection and staging for patients with pancreatic cancer as well as endoscopic ultrasound (EUS). Contrast-enhanced CT is regarded as primary approach for the diagnosis and resectability evaluation. MRI is an alternative choice and is superior to CT when evaluating ductal anatomy with MRCP [17]. Besides, MRI is superior to detect the liver metastases compared to CT with higher sensitivity [18]. Recently, PET-MRI has been reported to have equal efficacy in resectability evaluation for the patients with PDAC [19]. Yamada et al. showed that EUS combined with elastography (EG) had better diagnostic performance in evaluating vascular invasion for PDAC compared to CT [20]. In addition, Ehrlich et al. also demonstrated that for patients with borderline resectable pancreatic cancer and locally advanced pancreatic cancer, EUS-FNA (fine-needle aspiration) has the potential to ensure the diagnose as well as local resectability accurately and suggested it as a routine approach for PDAC patients [21]. However, a recent meta-analysis indicated that CT might be superior to EUS in resectability evaluation; so a controversy about EUS application still remains and more high-quality clinical trials need to be conducted in the future to achieve more high-level evidence [22,23,24].

### 1.2. Neoadjuvant and Adjuvant Therapy 

The impact of adjuvant chemotherapy to improve survival after resection of pancreatic cancer has been undoubtedly be proven during the last two decades, namely by the ESPAC study group as well as the PRODIGE consortium who continuously developed standards for adjuvant treatment by conducting large multicenter RCTs [25,26,27,28].

The latest of these studies reported median survival times of 30 and 54 months, respectively, as well as a 5-year survival of 30% which shows the essential need for adjuvant systemic treatment after pancreatic cancer resection [26,28]. This has ultimately been adopted in national and international guidelines [29]. 

Today there is a worldwide trend to increase the proportion of patients receiving neoadjuvant therapy. While neoadjuvant therapy is inevitable in locally advanced pancreatic cancers to achieve a chance of conversion surgery afterwards, its use in borderline-resectable and especially resectable pancreatic cancer is currently still based on weak evidence, although observational and a limited number of randomized controlled trials suggest its benefit when borderline resectable disease is considered.

Yet, the main dilemma remains the selection of patients for neoadjuvant treatment and the selection of the specific treatment protocol. Neoadjuvant chemotherapy alone or in combination with radiotherapy is widely used in numerous varying protocols on one hand, on the other hand these protocols are often based on institutional or national preferences and—in contrast to adjuvant protocols—no standards are set on the basis of high-quality evidence [30,31].

Briefly, the debate on upfront surgery versus neoadjuvant treatment still remains. The Dutch Randomized Phase III PREOPANC Trial demonstrated that there was no significant difference in overall survival benefit between the preoperative chemoradiotherapy and upfront surgery for resectable and borderline resectable pancreatic cancer [32]. Given the observation that upfront surgery combined with adjuvant therapy can attain an average 19% five-year overall survival which increases up to 50% in prognostically favorable subgroups neoadjuvant therapy is still far from being the standard based on high-level evidence [33]. If neoadjuvant therapy is chosen, another unsolved question is the need for additional adjuvant therapy after resection. A recently published study pooling observational data of 520 patient after induction FOLFIRINOX treatment and consecutive resection showed that an additional adjuvant protocol did not generally show any benefit but may be recommended for pathologically lymph-node positive patients [34]. A phase 2 Randomized Clinical Trial discovered that perioperative chemotherapy did not significantly improve two-year overall survival for resectable PDAC whereas may increase actual resectability rates—an observation which is certainly explained by a selection effect during neoadjuvant treatment [35]. All in all, adjuvant and especially neoadjuvant treatment are currently in a dynamic state and numerous studies are ongoing.

## 2. Surgery 

### 2.1. Standard Resection

Pancreaticoduodenectomy (PD) has been widely applied since in 1940, Whipple reported the classical procedure including distal gastrectomy and total duodenectomy and although this approach has been modified in some steps it is still basically similar to what is performed today [36]. 

PD includes a standardized lymphadenectomy along the right side of the vascular structures (porto-mesenteric veins, superior mesenteric artery, celiac axis) and the hepatoduodenal ligament. Nowadays, PD is routinely performed under preservation of the pylorus as recent studies have confirmed that pylorus preservation does not have any disadvantages compared to pylorus resection or classical Whipple procedures in terms of functional (especially regarding delayed gastric emptying) and oncological outcomes unless the tumor extends towards the pylorus, which then—unquestionably—requires resection of the distal stomach. 

For tumors of the body and tail of the pancreas, a distal pancreatectomy and splenectomy with respective lymphadenectomy from the left side of the vascular structures is mandatory.

In case of unfavorable location of the tumor in the center of the pancreas or synchronous multiple PDAC, a total pancreatectomy and splenectomy may be required. Regarding all resection techniques, it is of the utmost importance to achieve a radical (R0) resection status. This can best be achieved by a complete dissection of all lymphatic and soft tissue along the arterial structures to reduce the risk of remaining microscopic tumor persistence and early recurrence.

### 2.2. Specific Techniques 

#### 2.2.1. Artery First Approach 

The core principle of this procedure is to identify the SMA early at the origin of the aorta and the approach has been described for different ways of access to the artery [37,38]. The idea of the approach to evaluate any potential tumor adherence to the SMA at the beginning of the operation and either stop resection or plan an arterial resection if required and indicated. After exposing the SMA from the left-sided access (opening Treitz ligament) a Kocher maneuver is required to expose the anterior surface of the inferior vena cava and the aorta with an early identification of the left renal vein and the origin of the SMA. After the accurate dissection along the SMA is finished, the soft tissue between the SMA and the celiac trunk should also be removed. By this procedure, a very controlled and radical resection on the right side of the arterial axis (SMA/celiac trunk) is achieved, while the autonomous nerves on the left side of the arteries are spared to reduce the incidence of postoperative diarrhea. For the radical resection of pancreatic head tumors which involve the posterior and right side of the SMA, the artery first technique is beneficial and recommended. A recent meta-analysis indicated that the SMA artery first approach can decrease the overall complication rate (OR 0.62, 95% 17 CI 0.47 to 0.81, *p* = 0.001) and reduce blood loss (WMD −264.84, 95% CI −336.1 to 18 −193.58, *p* < 0.001) compared to the normal procedure in pancreaticoduodenectomy and attain an increased R0 resection rate (OR 2.92, 95% CI 1.72 to 4.96, *p* < 0.001) and three-year OS (OR 2.15, 95% CI 1.34 to 3.43, *p* = 0.001) showing that the artery first approach can have superior clinical outcomes [39]. Until now, many different artery first approaches have been developed, such as the posterior approach, the right/medial uncinate approach, the inferior infracolic or mesenteric approach or the hanging maneuver [40]. This underlines the importance of paying attention to the status of the SMA and achieving an increased R0 rate through the meticulous dissection of the right margin of the SMA.

#### 2.2.2. Uncinate Process First

The Uncinate first approach describes a modified technique of resection along of the right margin of the SMV and SMA through a special method. This approach includes the division of the proximal jejunum and translocation of the first jejunal loop before other steps of dissection. Afterwards, the pancreatic head is dissected retrogradely and finally leading to the transection of the pancreas at its neck [41]. The first step of the approach is to open Treitz ligament from the left side of the mesenteric root after the Kocher maneuver with wide mobilization of the duodenum. After division and skeletonizing the first jejunal loop, this is then pulled through to the right side of the mesenteric root and resection can be continued as described above. When using this method, there is no need to use tunneling to transect the pancreas above the portal vein for the specimen is usually already mobilized extensively. Through the retrograde approach, the resection may be more radical due to a clear visualization of the medial resection margin throughout the entire preparation and both superior mesenteric vessels, arteries and veins are clearly seen which may reduce blood loss. Hence, it is recommended as an additional technique in modern pancreatic surgery. Recently, it was demonstrated that also laparoscopic uncinate first approach is a feasible method for pancreatic head neoplasms with high lymph node harvests (19.3 vs. 13.9 (*p* = 0.03)) and no significant difference in R0 resection, operative time and median length of stay compared to laparoscopic classical approach [42]. Zhang et al. reported that laparoscopic pancreaticoduodenectomy (LPD) combined with the uncinate process first approach improved the laparoscopic resection technique with low risk of postoperative complications and high rate of curative resection [43]. Wang et al. described that LPD with uncinate process first reduced the operative time, decreased the bleeding amount during the operation and protected the variant hepatic artery suggesting that it is safe and feasible to conduct LPD together with uncinate first approach [44]. Additionally, a recent comparative study displayed that LPD with the uncinate process-first approach was feasible compared to traditional pancreatic surgery for this new technique can achieve less blood loss and a shorter first flatus time together with diet start time [45].

#### 2.2.3. The TRIANGLE Operation

**Figure 2:**
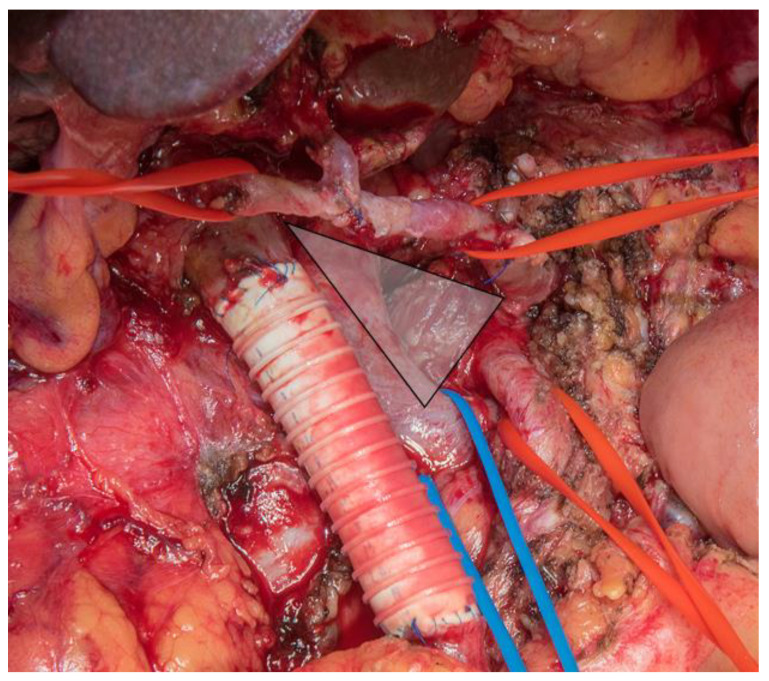
Intraoperative view after radical resection in pancreatic cancer (TRIANGLE operation). Porto-mesenteric vein resection and reconstruction with ringed allograft, dissection of all soft tissue (grey triangle) between celiac axis and superior mesenteric artery (red tapes) as well as the replaced mesenterico-portal vein. Blue tape: left kidney vein.

The TRIANGLE operation aims to develop a novel method for the patients with locally advanced pancreatic cancer after the neoadjuvant therapy and was described in 2017 [46]. The rationale of this procedure is the observation that after neoadjuvant therapy conventional imaging fails to differentiate between actual tumor encasement or abutment and only fibrotic residual tissue mainly to the arterial structures. Therefore, the technique comprises dissection of all soft tissue along the CA, SMA, SMV, and PV in association with a radical tumor removal. During the resection process, if must be proven that the specific periarterial tissue does not include viable tumor by frozen section; afterwards a radical artery-sparing approach can be conducted. This results in an anatomic triangle bordered by the SMA, CA, and portal vein revealed by the dissection and finally resection indicating the comprehensive removal of all soft tissue contained within these borders—usually fibrotic, neural, and lymphatic tissue (Figure 2). It is essential for the artery to be reached on the adventitial layer which opens longitudinally and allows to carry out the lymphadenectomy and soft tissue removal of the respective area. Above all, this technique allows patients after neoadjuvant therapy have the chance to attain a comprehensive tumor removal. Furthermore, the major advantage is the avoidance of arterial resection and reconstruction.

Furthermore, the major advantage is the avoidance of arterial resection and reconstruction. However, when required, the TRIANGLE operation can be combined with and arterial resection and reconstruction, a venous resection is frequently required in this situation. Rosso et al. described that the “triangle operation” for borderline resectable pancreatic head cancer was safe and efficient [47]. 

#### 2.2.4. Venous Bypass First

One of the most challenging procedures during pancreatectomy can arise when venous infiltration of the portal/superior mesenteric vein axis is basically possible but hampered by large collateral vessels which implies that preparation may take a rather long time with the consecutive need for a long clamping time towards the small bowel with venous congestion [48]. In such situations, including cavernous transformation of the portal vein, a new surgical technique called “venous bypass graft first” is the procedure of choice [49,50]. The idea of this procedure is to create an initial venous bypass graft placement between the superior mesenteric vein or its tributaries and the portal vein in order to avoid bleeding as well as venous congestion of the small bowel. If the portal vein is not accessible in the hepatoduodenal ligament or liver hilum, this bypass can be performed between superior mesenteric vein and inferior cava vein after the Kocher/Cattel-Braasch maneuver is completed before proceeding with the resection of the pancreatic head. As cavernous transformation of the portal vein is caused by a complete portal/superior mesenteric vein occlusion; otherwise, it is an unsolved obstacle for resection, the step-by-step pancreatic head resections with a ‘venous bypass graft first’ approach should be carried out to overcome this problem. The approach includes preoperative assessment of the superior mesenteric and portal vein, exploration, and identification of venous vessels suitable for a graft placement. By this technique, a continuous porto-venous inflow to the liver during the resection phase is ensured if performed as a mesenterico-portal bypass. If this is not directly possible, at least a severe venous congestion of the small bowel can be avoided. in cases of temporary mesenterico-caval shunting and final restoration of the portal vein inflow reconstruction to the portal vein after completed tumor resection. 

#### 2.2.5. Periarterial Divestment

Due to the increasing application of neoadjuvant therapy in PDAC, especially in locally advanced disease, surgical strategies and concepts have gradually changed as well as resection techniques, especially for cases which have been down-staged or shown a stable disease. It still remains controversial whether it is mandatory to perform arterial resection for arterial involvement in pancreatic cancer. An alternative approach has been described as the “periarterial divestment” technique [51,52]. This technique comprises a radical tumor clearance without arterial resection instead. Because of the inaccuracy of detection of true arterial involvement and true arterial invasion through current imaging methods, operative exploration should be performed. 

The technique of periarterial divestment describes the sub-adventitial dissection in the layer between the arterial wall and remnant tumor/fibrous tissue which allows a radical removal without an arterial replacement. All in all, ‘artery first’ approach, ‘uncinate process first’, ‘triangle operation’, ‘venous bypass first’, and ‘periarterial divestment’ are complementary techniques in pancreatic cancer surgery. These mainly vessel-oriented technical approaches of pancreatic head resection allow removal of all putatively tumor-infiltrated soft tissue with the utmost aim for an improved R0 resection rate [53].

### 2.3. Vascular (Venous and Arterial) Resection

#### 2.3.1. Venous Resection 

Vascular resection, especially for venous resection has now been widely applied with pancreaticoduodenectomy in selected patients. The earliest surgery focusing on the superior mesenteric vein (SMV) was reported by Moore in 1951 during pancreatic surgery [54]. Afterwards, en bloc pancreatoduodenectomy with vein resection was described by Fortner and indicated that the technique is safe and favorable [55]. Venous resections have been modified and refined to be a routine surgical procedure in high volume centers [56,57]. It is possible to perform vein resection in patients with PDAC during all types of pancreatic surgery including pancreaticoduodenectomy, distal, or total pancreatectomies. The ISGPS classified mesentericoportal vein resections into four groups which was mainly considered by the approaches of resection and reconstruction [12]. Regarding outcomes of these techniques, vascular resection along with multiple treatments is beneficial for the patients with pancreatic cancer especially in the long-term overall survival. [58]. Several observational studies [59,60] demonstrated that neoadjuvant systematic chemotherapy can lead to increased radical resection chances for patients with complex tumor-vessel anatomy. The 2019 French Recommendations for the Vascular Resection for Pancreatic Cancer [60] has suggested that neoadjuvant treatment should be applied in case of venous tumor involvement followed by pancreatectomy with venous resection and can potentially be curative for the respective patients. It is unquestionable that venous resection during PD must also aim to obtain negative resection margins, while the reported effects on survival remain controversial [61]. A meta-analysis showed that pancreatectomy combined with venous resection needed longer operative time and had increased perioperative blood loss compared to the group of pancreatectomy without venous resection [62]. Patients with venous resection attained reduced R0 rates. There was no significant difference in postoperative complications between the two groups. In terms of survival, patients with venous resection had lower one-, three-, and five-year survival. The most recent meta-analysis [63] described that patients with pancreaticoduodenectomy plus venous resection seemed to attain a larger tumor size, positive lymph nodes and R1 resection rates and higher 30 day mortality. However, there was no significant difference in rates of total complications. In terms of long-term outcomes, patients with venous resection had lower one-year overall survival (OS), three-year OS, and five-year OS. A retrospective study [64] revealed that patients during pancreatic resection with venous vascular resection attained higher morbidity, lower five-year disease-free survival (7% and 20%, *p* = 0.018) and five-year disease-specific survival (19% and 35%, *p* = 0.42). Controversially to the reported impaired survival after venous resection, a recent propensity score-matched analysis [65] showed similar survival in pancreaticoduodenectomy with venous resection and pancreaticoduodenectomy alone groups after adjustment for baseline characteristics. A Japanese study [66] described the feasibility of venous resection and—in combination with adjuvant therapy—favorable outcomes reaching a 30-month median survival time in borderline resectable patients. This underlines the need to perform a venous resection whenever required to achieve negative resection margins and not to compromise radicality by avoidance of vascular resection and reconstruction. However, the effects of the various treatment options—including neoadjuvant therapy—in this setting require further evaluation and more high-level studies need to be conducted in the future.

#### 2.3.2. Artery Resection 

**Figure 3:**
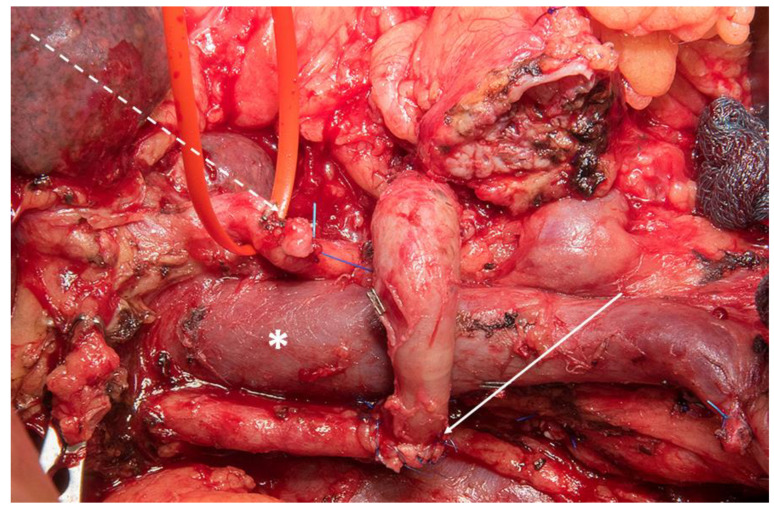
Example of splenic artery transposition on an aberrant right hepatic artery after resection of the aberrant hepatic artery due to tumor infiltration. Proper left hepatic artery with red tape and stump of the gastroduodenal artery (broken white arrow); portal vein (white asterisk); transposed splenic artery with end-to-end anastomosis on the aberrant right hepatic artery.

**Figure 4:**
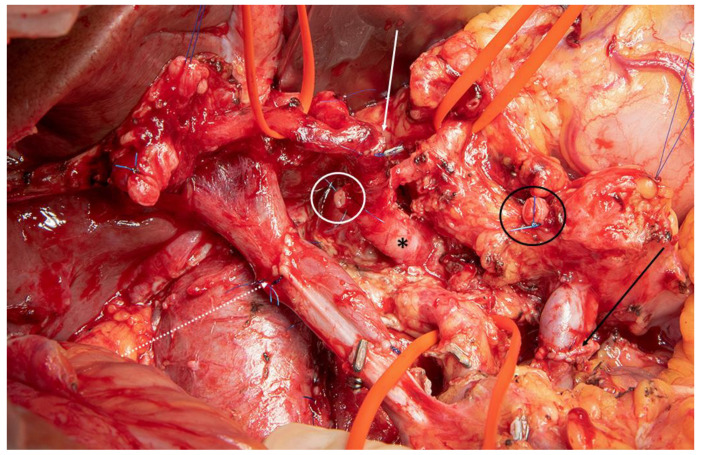
Intraoperative view after combined arterial and venous resection during partial pancreato-duodenectomy. Resection of the common hepatic artery (white circle) and reconstruction by splenic artery transposition with end-to-end anastomosis (white arrow) on the proper hepatic artery (upper left red tape). Distal splenic artery stump (black circle) below the pancreatic cut margin; black asterisk: celiac axis; end-to-end reconstruction of the superior mesenteric/portal vein (dotted white arrow) and splenic vein on inferior mesenteric vein (black circle); upper right red tape: left gastric artery; lower middle red tape: superior mesenteric artery.

In the 1950s, arterial resection was initially described during abdominal surgery by Appleby on resection of the celiac axis during extended gastrectomy including distal pancreatectomy [67]. In contrast to vein resection, artery resection is more debatable for its increased morbidity and mortality and mostly considered as an individual decision in selected patients [68]. However, the modified Appleby procedure which implies distal pancreatectomy, splenectomy, and celiac axis resection under preservation of the stomach has been shown to be beneficial for the patients with advanced tumors of the pancreatic body and tail [69]. This procedure can achieve median survival times of at least 18 months when combined with a multimodal treatment concept and is gaining increasing acceptance today [70]. Furthermore, during recent years, the techniques of replacement applied for the hepatic artery or the superior mesenteric artery have been improved and procedures such as splenic artery use have been described for restoration of hepatic or small-intestine perfusion (Figure 3 and Figure 4) [71]. Oba et al. confirmed that arterial resection it is more likely to attain preferable long-term outcome after the application of preoperative neoadjuvant treatments [72]. A Japanese study reported that patients with distal pancreatectomy plus celiac axis resection who underwent preoperative therapy achieved better one-, two-, and five-year overall survivals (100%, 90%, and 78.8%) than those who underwent upfront surgery (77.9%, 51.5%, and 26.7%; *p* < 0.0001) [73]. A recent meta-analysis showed that patients undergoing pancreatic surgery with artery resection had a greater risk of postoperative mortality (RR: 4.09, *p* < 0.001), morbidity (RR: 1.4, *p* = 0.01) and worse three-year survival [74]. Regarding specific complications and outcomes, the postoperative complications and the length of hospital stay and non-R0 rate were not significantly different compared to those without artery resection. A single-center cohort study reported that pancreatectomy with artery resection can attain better one-, three-, and five-year survival rates compared to palliation for patients with LAPC [75]. Another recent study covering nearly 40 years of experience showed that any type of arterial resection was performed at a frequency of 6% (44/730 patients) and confirmed the safety and efficacy of these operations for patients with locally advanced pancreatic cancer, additionally suggesting preoperative therapy with artery resection as a useful concept for locally advanced pancreatic cancer [76]. An important aspect in selecting patients properly and gaining sufficient surgical experience to safely perform such procedure which has recently been shown in two large series that demonstrated the impact of the surgical learning curve in two single center collectives of 111 and 195 patients, respectively [77,78].

### 2.4. Multivisceral Resection

In addition to vascular resections, also multivisceral resections have been applied increasingly nowadays to attempt to achieve margin-negative resection. Previous studies indicate that pancreaticoduodenectomy with multivisceral resection is associated with increased morbidity and potentially mortality with conflicting results in terms of oncologic outcomes [79,80,81]. A systematic review suggested that multivisceral pancreatectomies was safe and feasible in selected patients [82]. A case-matched study showed that multivisceral distal pancreatectomy was able to achieve radical tumor removal providing beneficial survival outcomes [83]. Furthermore, a single center analysis demonstrated that multivisceral resection in pancreatic surgery was suitable for locally advanced pancreatic carcinoma of the body and/or tail [84], comparable results were achieved in a current multi-center publication, proving that distal pancreatectomy with multivisceral resection is viable in order to obtain free margins which is the key to achieve long-term survival [85].

### 2.5. MIS/Robotic Surgery

With the rapid development of technology, minimal invasive pancreatic surgery has been popularly applied worldwide. The first laparoscopic pancreatectomy was reported in 1996 while the first robotic pancreatic resections were described in 2003 [86,87]. A recent international evidence-based guideline on minimally-invasive pancreatic surgery demonstrated that open, laparascopic and robotic pancreatic surgery all have their own aspects in treating patients with pancreatic diseases and it is quite possible to achieve promising clinical outcomes by applying these advanced technologies [88]. An international consensus statement on robotic pancreatic surgery showed that robotic pancreatic surgery is safe and feasible compared to open pancreatic surgery [89]. Another international expert consensus on laparoscopic pancreaticoduodenectomy (PD) also showed that laparoscopic pancreaticoduodenectomy was safe and effective for experienced surgeons [90]. Furthermore, a current network meta-analysis indicated that laparoscopic PD and robotic PD had a reduced length of hospital stay, operative bleeding and overall complications while on the other hand achieving a similar number of retrieved lymph nodes, tumor-free resection margins, clinically relevant postoperative pancreatic fistula, severe postoperative complications [91]. Besselink et al. demonstrated that minimally invasive distal pancreatectomy (DP) is technically safe, whereas oncological feasibility needs to be evaluated carefully. With respect to minimally invasive PD, some advantages have been shown in comparison to open PD [92]. However, due to a limited level of evidence, this has to be regarded with care, but minimally-invasive PD could be beneficial for selected patients with better short-term clinical outcomes. Without doubt, there is a strong need for more high-quality trials to confirm potential advantages of minimally invasive pancreatic surgery.

#### 2.5.1. Laparoscopic and Robotic Distal Pancreatectomy

The latest study indicated a shorter length of hospital, less delayed gastric emptying, higher rates of postoperative pancreatic fistula in minimally invasive distal pancreatectomy in contrast to open distal pancreatectomy [93]. The DIPLOMA study indicated that minimally invasive distal pancreatectomy attained less median blood loss, shorter hospital stay and less lymph node retrieval [94]. Furthermore, the LEOPARD randomized controlled trial proved that the less operative blood loss and the rate of delayed gastric emptying. However, longer operation times (217 vs. 179 min, *p* = 0.005) were observed in minimally invasive distal pancreatectomy [95]. A multicenter study described no significant differences in the incidence of clinically relevant postoperative pancreatic fistula in robotic distal pancreatectomy in contrast with open pancreatectomy [96]. 

#### 2.5.2. Laparoscopic and Robotic Pancreatoduodenectomy

A recent study showed that laparoscopic pancreaticoduodenectomy had lower blood loss, longer operative time. However, there were no obvious differences among 90-day overall mortality, Clavien-Dindo 3 complications and postoperative length of hospital stay in contrast to open surgery [97]. This meta-analysis included—among two other studies—the LEOPARD-2 randomized controlled phase 2/3 trial which reported more complication-related deaths in the laparoscopic group compared to open pancreaticoduodenectomy with no obvious difference in time to functional recovery between the two groups and thereby weakened the conclusion that laparoscopic pancreaticoduodenectomy is potentially harmful [98]. Yet, this procedure is probably only feasible in highly-specialized centers with a respective high case load. However, the level of the current evidence focusing on minimal invasive pancreaticoduodenectomy may be too low, hence, more high-quality studies need to be carried out to enhance the future evidence, as especially for robotic procedures no randomized controlled trials are available to date.

## 3. Conclusions and Future Perspective

Over the past decades, surgical therapy for pancreatic cancer has been changing and developing rapidly allowing extended resections and improved complication. Given the advanced technology and comprehensive strategies, approaches of curative resections have improved as well as the quality of perioperative management. As a result, the mortality rate after pancreatic surgery has reduced obviously to a current rate of less than 5% in specialized centers. Although centralization has not become reality in all countries around the world, this should be the benchmark and especially advanced pancreatic surgery should be clearly limited to high-volume centers. Combined with multimodal treatment, pancreatic surgery allows to improve the quality of life and long-term survival for patients in different stages of pancreatic cancer. In terms of surgical techniques, open, laparoscopic and robotic procedures all will exert their own merit in their particular field to achieve benefit for the patients at the greatest extent.

## Figures and Tables

**Table 1:** International consensus of classification of BR PDAC based on anatomical definition using CT imaging including coronal and sagittal sections [7].

Category	Anatomical Feature
Resectable: R	SMV/PV: no tumor contact or unilateral narrowing
	SMA, CA, CHA: no tumor contact
Borderline resectable: BR	Subclassified according to SMV/PV involvement alone or arterial invasion
BR-PV (SMV/PV involvement alone)	SMV/PV: tumor contact 180 or greater or bilateral narrowing/occlusion, not exceeding the inferior border of the duodenum.
	SMA, CA, CHA: no tumor contact/invasion
BR-A (arterial involvement)	SMA, CA: tumor contact of less than 180 without showing deformity/stenosis
	CHA: tumor contact without showing tumor contact of the PHA and/or CA.
Unresectable: UR	Subclassified according to the status of distant metastasis
Locally advanced: LA	SMV/PV: bilateral narrowing/occlusion, exceeding the inferior border of the duodenum
	SMA, CA: tumor contact/invasion of 180 or more degree #.
	CHA: tumor contact/invasion showing tumor contact/invasion of the PHA and/or CA.
	AO: tumor contact or invasion.
Metastatic: M	Distant metastasis $.

#: In cases with CA invasion of 180 or more without involvement of the aorta and with intact and uninvolved gastroduodenal artery thereby permitting a distal pancreatectomy with en bloc celiac axis resection (DP-CAR), some members prefer this criteria to be in the BR-A category. $: including macroscopic para aortic and extra abdominal lymph node metastasis.

## Data Availability

Not applicable.

## Abbreviations

AHPBA	American Hepato-Pancreato-Biliary Association
SSO	Society of Surgical Oncology
SSAT	Society for Surgery of the Alimentary Tract
ECOG	Eastern Cooperative Oncology Group
BACAP	The National Anatomo-Clinical Database on Pancreatic Adenocarcinoma
SMV	superior mesenteric vein
PV	portal vein
SMA	superior mesenteric artery
CA	celiac artery
CHA	common hepatic artery
PHA	proper hepatic artery
LAPC	locally advanced pancreatic cancer
WMD	weighted mean difference
OR	odds ratio
CI	confidence interval
